# Riboregulation in the Major Gastric Pathogen *Helicobacter pylori*

**DOI:** 10.3389/fmicb.2021.712804

**Published:** 2021-07-16

**Authors:** Alejandro Tejada-Arranz, Hilde De Reuse

**Affiliations:** ^1^Unité Pathogenèse de Helicobacter, CNRS UMR 2001, Département de Microbiologie, Institut Pasteur, Paris, France; ^2^Université de Paris, Sorbonne Paris Cité, Paris, France

**Keywords:** small RNAs, antisense RNAs, virulence, phase variation, *Helicobacter pylori*, post-transcriptional regulation

## Abstract

*Helicobacter pylori* is a Gram-negative bacterial pathogen that colonizes the stomach of about half of the human population worldwide. Infection by *H. pylori* is generally acquired during childhood and this bacterium rapidly establishes a persistent colonization. *H. pylori* causes chronic gastritis that, in some cases, progresses into peptic ulcer disease or adenocarcinoma that is responsible for about 800,000 deaths in the world every year. *H. pylori* has evolved efficient adaptive strategies to colonize the stomach, a particularly hostile acidic environment. Few transcriptional regulators are encoded by the small *H. pylori* genome and post-transcriptional regulation has been proposed as a major level of control of gene expression in this pathogen. The transcriptome and transcription start sites (TSSs) of *H. pylori* strain 26695 have been defined at the genome level. This revealed the existence of a total of 1,907 TSSs among which more than 900 TSSs for non-coding RNAs (ncRNAs) including 60 validated small RNAs (sRNAs) and abundant anti-sense RNAs, few of which have been experimentally validated. An RNA degradosome was shown to play a central role in the control of mRNA and antisense RNA decay in *H. pylori*. Riboregulation, genetic regulation by RNA, has also been revealed and depends both on antisense RNAs and small RNAs. Known examples will be presented in this review. Antisense RNA regulation was reported for some virulence factors and for several type I toxin antitoxin systems, one of which controls the morphological transition of *H. pylori* spiral shape to round coccoids. Interestingly, the few documented cases of small RNA-based regulation suggest that their mechanisms do not follow the same rules that were well established in the model organism *Escherichia coli*. First, the genome of *H. pylori* encodes none of the two well-described RNA chaperones, Hfq and ProQ that are important for riboregulation in several organisms. Second, some of the reported small RNAs target, through “rheostat”-like mechanisms, repeat-rich stretches in the 5′-untranslated region of genes encoding important virulence factors. In conclusion, there are still many unanswered questions about the extent and underlying mechanisms of riboregulation in *H. pylori* but recent publications highlighted original mechanisms making this important pathogen an interesting study model.

## Introduction

**FIGURE 1:**
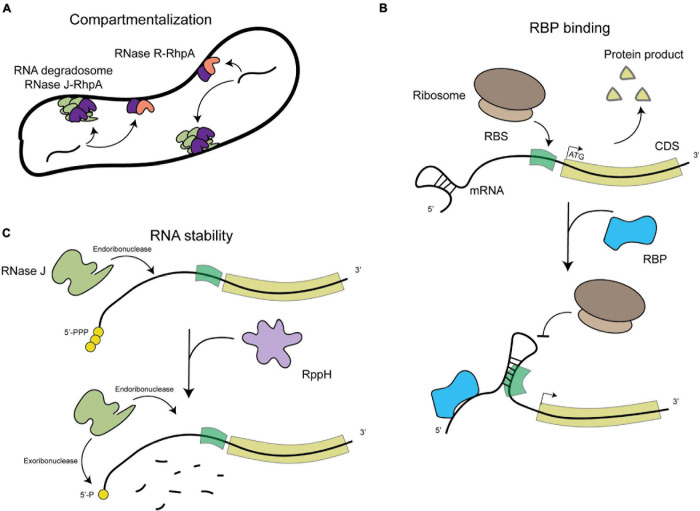
Schematic representation of the general levels of post-transcriptional regulation in *H. pylori*. **(A)** Compartmentalization of RNA degradation, with the RNA degradosome composed of RNAse J (in green) and RhpA (in pink) localizing into foci at the inner membrane and the RNase R (in dark purple)-RhpA complex also binding the inner membrane. **(B)** RNA binding protein (RBP)-mediated regulation, where binding of an RBP (in blue) can cause a structural rearrangement in the RNA molecule that allows or prevents the expression of the proteins it encodes. **(C)** RNA degradation by both endo- and exoribonucleases, such as RNase J (in light green), which also depends on the phosphorylation state of the 5′-end of the mRNA molecules (RppH in light purple).

**FIGURE 2:**
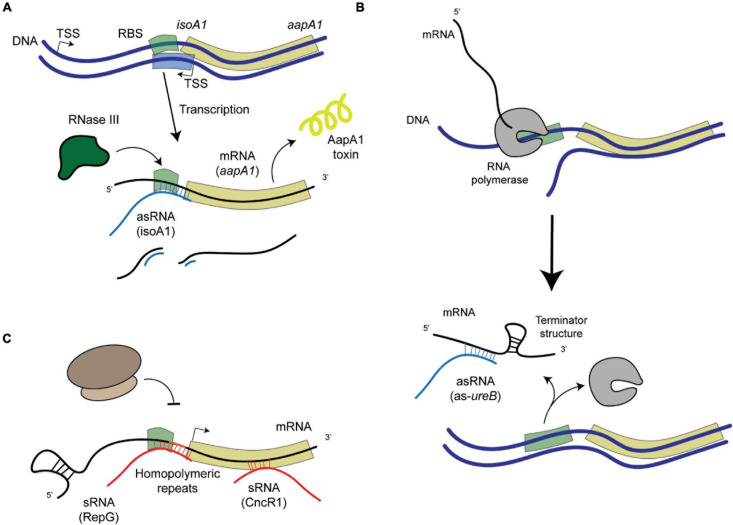
Schematic representation of examples of post-transcriptional regulation in *H. pylori*. **(A)** asRNA-mediated regulation that leads to the degradation of the mRNA-asRNA pair by an enribodonuclease such as RNase III, like in the case of the IsoA1-*aapA1* Toxin-Antitoxin system. **(B)** asRNA-mediated regulation that leads to the formation of a premature terminator sequence that prevents the full transcription of the target gene (as in the case of the 5′-*ureB* antisense). **(C)** sRNA-mediated regulation, where sRNAs (encoded elsewhere in the genome apart from their targets) can either bind homopolymeric repeats upstream from the gene (like RepG) or at different regions within the coding sequence (like CncR1).

*Helicobacter pylori* is a Gram-negative bacterium belonging to the epsilon-proteobacteria class recently proposed to be renamed as *Campylobacterota* (Waite et al., 2017). *H. pylori* is a microaerophilic and helical shaped microorganism that inhabits the stomach of half of the human population worldwide. *H. pylori* is transmitted between humans and generally acquired before the age of five. Infected individuals suffer from chronic gastritis that can remain asymptomatic throughout their lives in 85% of the cases or can evolve into a range of disorders including peptic ulcers or gastric adenocarcinoma, that is responsible for about 800,000 deaths every year worldwide (Robinson and Atherton, 2021). The severe pathologies associated to *H. pylori* infection, like cancer, generally occur after decades of chronic infection. *H. pylori* has evolved to persistently colonize the stomach, despite the harsh conditions of this hostile environment, such as a very low pH and constantly changing conditions, which indicates that it has a strong adaptation capacity. It was thus surprising to observe that *H. pylori* possesses few [only 16 (De la Cruz et al., 2017)] transcriptional regulators (Tomb et al., 1997). This is consistent with its small genome of only 1.67 Mb that encodes 1,576 open reading frames (ORFs) in the 26695 type strain and its unique human gastric niche. Given the reduced number of transcriptional regulators, post-transcriptional regulation has been proposed to play a major role in the control of gene expression in *H. pylori* (Pernitzsch and Sharma, 2012). *H. pylori* does not possess RNA chaperones like Hfq and ProQ, that are important factors in post-transcriptional regulation in many bacteria, including the model organism *Escherichia coli* (Quendera et al., 2020). This characteristic has been an impediment for rapid regulatory RNAs identification in *H. pylori* but it also hints to original mechanisms for post-transcriptional regulation, that we will address in this review and are summarized in Figures 1, 2.

## The Transcriptome of *H. pylori*

The seminal work by Sharma et al. (2010) revealed the complexity of the transcriptome of this bacterium. They mapped 1,907 transcription start sites (TSSs) in *H. pylori* strain 26695 and revealed that 87.5% of the genes are expressed from 337 primary operons, some of which contain additional internal TSSs. Surprisingly, they also detected massive antisense transcription with at least one antisense TSS associated to approximately 46% of all ORFs, including housekeeping genes like 28% of tRNAs and the 5′-leader regions of the 23S and 16S rRNA precursors. Interestingly, most known riboswitches are absent in *H. pylori*, with the exception of a predicted thiamine pyrophosphate riboswitch upstream of *pnuC*. Riboswitches are elements in an RNA molecule that can alter their structure in response to an environmental signal, such as a temperature shift or the presence of certain metabolites, and as a consequence regulate the translation or degradation of this RNA molecule. Even so, there are 337 untranslated regions (UTRs) that are long enough to accommodate other *cis*-acting regulatory RNA structures (Sharma et al., 2010).

In addition to tRNAs, rRNAs, transfer-messenger RNA (*tm*RNA, an RNA molecule in charge of ribosome unstalling), RNase P, 6S RNA and the signal recognition particle RNA (SRP RNA), Sharma et al. (2010) detected hundreds of candidate non-coding RNAs (ncRNAs). These ncRNAs are transcribed from intergenic regions (small RNAs or sRNAs), antisense to ORFs (antisense RNAs, asRNAs) and sense within ORFs. The expression of 60 of these intergenic ncRNAs was validated (visualized by Northern blot), and more than 900 asRNAs were detected, a few of them being validated (Sharma et al., 2010). It is interesting to note that, even if such massive amounts of asRNAs were first detected in *H. pylori*, other bacteria have since then been shown to have even more asRNAs (Georg and Hess, 2018). The validated and characterized ncRNAs are summarized in Table 1.

**TABLE 1:** List of non-coding RNAs (ncRNAs) validated (visualized by Northern blot) in *Helicobacter pylori*.

**ncRNA**	**sRNA or asRNA**	**Demonstrated target (when known)**	**Phenotypes regulated**	**Expression regulation**	**References**
*tm*RNA	–	Stalled ribosomes	Competence; oxidative and antibiotic stress tolerance	Induction under acidic conditions	Thibonnier et al. (2008)
6S RNA	–	RNA polymerase	Global regulator of transcription		Sharma et al. (2010)
SRP RNA	–	SRP	Membrane protein targeting		Sharma et al. (2010)
IsoA1	asRNA	*aapA1* (Type I TA system)	Growth and morphology (coccoid transition)	Repression by hydrogen peroxide	Sharma et al. (2010), Arnion et al. (2017), El Mortaji et al. (2020)
IsoA3	asRNA	*aapA3* (Type I TA system)	Growth		Sharma et al. (2010), Masachis et al. (2019)
23S asRNA	asRNA	23S-5S rRNA precursor			Iost et al. (2019)
IG-443	asRNA	antisense to *fliM*			Xiao et al. (2009)
IG-524	asRNA	antisense to *fumC*			Xiao et al. (2009)
RepG/HPnc5490	sRNA	*tlpB-hp0102*	Chemotaxis, LPS biosynthesis and resistance to antibiotics	As a function of growth phase, accumulates in coccoid forms	Sharma et al. (2010), Pernitzsch et al. (2014, 2021)
NikS/HPnc4160 (*IsoB*)	asRNA	*cagA, vacA*, *horF*, *hofC*, *horB*, *hopE*, *omp14*, *hpg27_1238, hp1227*, *hp0410*, *helpy_1262*	Bacterial internalization, colonization and epithelial barrier disruption, production of phosphorylated CagA in host cells	Phase-variable expression and regulated by NikR	Eisenbart et al. (2020), Kinoshita-Daitoku et al. (2021)
5′*ureB*-sRNA	asRNA	*ureB*	Urease activity	Expression at neutral pH, negatively regulated by the ArsRS TCS	Wen et al. (2011), Wen et al. (2013)
CncR1/HPnc2630	sRNA	*fliK*	Motility and adhesion to host cells	Growth phase regulated by HsrA (orphan response regulator)	Sharma et al. (2010), Vannini et al. (2014, 2016)
IsoA2	asRNA	*aapA2* (Type I TA system)			Sharma et al. (2010)
IsoA4	asRNA	*aapA4* (Type I TA system)			Sharma et al. (2010)
IsoA5	asRNA	*aapA5* (Type I TA system)			Sharma et al. (2010)
IsoA6	asRNA	*aapA6* (Type I TA system)			Sharma et al. (2010)
HPnc1200	asRNA	*rplU*			Sharma et al. (2010)
HPnc6270	asRNA	*cstA*			Sharma et al. (2010)
HPnc3200	asRNA	*hp0637*			Sharma et al. (2010)
HPnc3210	asRNA	*hp0637*			Sharma et al. (2010)
HPnc2240	asRNA	*Hp0488*			Sharma et al. (2010)
HPnc2250	asRNA	*Hp0488*			Sharma et al. (2010)
HPnc5970	asRNA	*Hp1116*			Sharma et al. (2010)
HPnc6000	asRNA	*Hp1116*			Sharma et al. (2010)
HPnc2450	asRNA	*Hp0513*			Sharma et al. (2010)
HPnc1470	asRNA	*Hp0357*			Sharma et al. (2010)
HPnc7450	asRNA	*HPr06*			Sharma et al. (2010)
HPnc3320	asRNA	*Hp0660*			Sharma et al. (2010)
HPnc1810	asRNA	*Hp0423*			Sharma et al. (2010)
HPnc7520	asRNA	*Hp1412*			Sharma et al. (2010)
HPnc6910	sRNA				Sharma et al. (2010), Rieder et al. (2012)

**Non-characterized sRNAs identified in Sharma et al. (2010)**					

HPnc6670, HPnc2090, HPnc2420, HPnc4590, HPnc7830, HPnc1810, HPnc6160, HPnc4870, HPnc7430, HPnc1880, HPnc5490, HPnc6670, HPnc2630, HPnc2640, HPnc0260 HPnc0270, HPnc1200, HPnc1820, HPnc7510, HPnc2250, HPnc5970, HPnc2440, HPnc3110, HPnc3200, HPnc3210, HPnc3830, HPnc3840, HPnc4510, HPnc4850, HPnc4870, HPnc5300, HPnc5580, HPnc6160, HPnc6270, HPnc6630, HPnc6620, HPnc7100, HPnc7230, HPnc7430, HPnc7770, HPnc7890, HPnc7830, HPnc4620, HPnc4630, HPnc0580, HPnc7830, HPnc0580, HPnc1990, HPnc1980, HPnc0470, HPnc0480, HPnc0490, HPnc2420, HPnc3560, HPnc5000, HPnc5130, HPnc5140, HPnc5310, HPnc5800, HPnc5810, HPnc6870, HPnc7300, HPnc7700, HPnc7720, HPnc7670, HPnc7680, HPnc0710, HPnc1070, HPnc3020, HPnc3880, HPnc4860, HPnc5960.					

*An online browser for the visualization of TSSs in *H. pylori* is available at http://www.imib-wuerzburg.de/research/hpylori/ (Bischler et al., 2015).*

## Tools to Study the Transcriptome of *H. pylori*

In the original description of the *H. pylori* transcriptome by Sharma et al. (2010), differential RNA sequencing (dRNA-seq) technologies were used to sequence and define TSSs, and such techniques have been further optimized since then (Bischler et al., 2015). First, in order to detect the widest range of TSSs, RNA was extracted from *H. pylori* grown under different conditions. Then prior to sequencing of cDNA libraries, RNAs were treated or not with terminator exoribonuclease. This enzyme specifically degrades 5′-P (monophosphate) RNA molecules [that usually result from processing of transcripts by ribonucleases (RNases)] but not 5′-PPP (triphosphate) RNA molecules (that result from transcription), thus allowing the detection of TSSs. Furthermore, the authors developed an online browser for the visualization of TSSs in *H. pylori*^1^ (Bischler et al., 2015).

As mentioned earlier, the main *E. coli* RNA-binding proteins (RBPs) participating in ncRNA-mediated regulation of gene expression, Hfq and ProQ, are absent in *H. pylori* (Quendera et al., 2020). Only a homolog of the RBP CsrA (Carbon storage regulator A) has been identified (Barnard et al., 2004). However, the complexity of the transcriptome suggests that other mechanisms and RBPs might be involved in post-transcriptional regulation in this organism.

Tools have been developed in recent years to identify novel RNA-RBP complexes in *H. pylori* (Rieder et al., 2012). One of these approaches was based on the purification of aptamer-tagged sRNAs by chromatography to identify their protein binding partners by mass spectrometry (Rieder et al., 2012). Complementarily, a method to FLAG-tag putative RBPs, co-immunoprecipitate them and sequence the associated RNA molecules was developed. Such tools permitted the detection of interactions between the S1 ribosomal protein and some mRNAs and sRNAs in *H. pylori*, as well as the identification of protein HP1334 as a binding partner of the abundant HPnc6910 sRNA (Rieder et al., 2012).

## The Membrane-Associated RNA Degradosome Regulates Antisense RNA Abundance

Despite progress in the detection of RNA species and their putative protein partners, nothing is known about the subcellular distribution of these elements in *H. pylori*. Different RNA molecules from the transcriptome of *E. coli* have been shown to display specific subcellular localization patterns, being targeted to the membrane, the cellular poles or distributed in the cytosol (Kannaiah et al., 2019). This likely plays a role in localizing their protein products and in regulating their expression and stability (Irastortza-Olaziregi and Amster-Choder, 2020). Bacterial RNA degradosomes, that are composed of at least one RNase and one DEAD-box RNA helicase protein that unwinds the target RNA, are central in the control of RNA decay and maturation (Tejada-Arranz et al., 2020a). The membrane localization of RNA degradosomes has been linked to the rate of degradation of different categories of transcripts including regulatory sRNAs (Moffitt et al., 2016), indicating a compartmentalization of RNA degradation and maturation that plays a role in transcriptome dynamics in prokaryotes. In *H. pylori*, we showed that the RNA degradosome, composed of the essential RNase J protein and of RhpA, the sole DEAD-box RNA helicase of this bacterium (Redko et al., 2013; El Mortaji et al., 2018), is compartmentalized at the inner membrane where it is assembled into foci whose formation is regulated and likely represent RNA degradation hubs (Tejada-Arranz et al., 2020b; Figures 1A,C). In addition, the 3′–5′ exoribonuclease RNase R was also associated to the *H. pylori* inner membrane (Tejada-Arranz et al., 2021; Figure 1A). The development of imaging techniques [reviewed in Fei and Sharma (2018)] for the study of the localization of RNA molecules in *H. pylori* is needed in order to assess how the transcriptome is distributed in *H. pylori* cells.

In *H. pylori*, we found that RNase J, the main RNase of its minimal RNA degradosome (Redko et al., 2013), is able to degrade many asRNAs (as well as mRNAs), with about 80% of them being upregulated more than 2-fold in an RNase J-depletion strain, and approximately 50% being regulated more than 4-fold (Redko et al., 2016). This suggests that a major level of regulation of the amount of these asRNAs relies on RNA degradosome-mediated degradation. Interestingly, sRNAs were found not to be preferred targets of this enzyme and are likely regulated through other mechanisms.

## Regulation by RNA-Binding Proteins (RBPS)

The only RBP that has been extensively studied in other bacteria and that is present in *H. pylori* is CsrA (Quendera et al., 2020). This protein is necessary for full *H. pylori* motility and survival under oxidative stress conditions. Accordingly, a strain lacking CsrA is defective for virulence in a mouse model (Barnard et al., 2004). More recent studies have found the reduced motility of a Δ*csrA* strain to be associated with a defect in flagellar assembly due to a reduced expression of FlaA and FlaB (Kao et al., 2014) and to the dysregulation of the expression of a putative glycosyltransferase (Kao et al., 2017). In *H. pylori*, no CsrB/D-like sRNAs, which regulate CsrA activity in other organisms, were identified and thus the regulation of the activity of CsrA is not known in this organism. Nevertheless, the role of CsrA in asRNA- and sRNA-mediated regulation remains to be investigated. Other RBPs have been found in *H. pylori*, like the glycolytic enzyme aconitase that was shown to be important for full motility, oxidative stress response and lysozyme resistance (Austin and Maier, 2013; Austin et al., 2015; see Figure 1B).

A homolog of the RNA pyrophosphohydrolase, RppH, was also characterized in *H. pylori*. This enzyme targets the 5′-end of both mRNAs and sRNAs in the cell and cleaves the 5′-triphosphorylated end, yielding a 5′-monophosphorylated molecule that is targeted for degradation (Bischler et al., 2017; Figure 1C). In addition, mutants in this protein are more susceptible to hydrogen peroxide (Lundin et al., 2003) and are less able to invade gastric adenocarcinoma cells (Liu et al., 2012).

## Non-Coding RNA-Mediated Regulation of Gene Expression

As mentioned above, the transcriptome of *H. pylori* contains >900 asRNA molecules and 60 validated sRNAs, with only one predicted riboswitch. Overall, there is little information about the molecular mechanisms by which asRNAs and sRNAs regulate gene expression and how their expression is regulated in *H. pylori*. Generally, these ncRNAs act by base-pairing with their target mRNAs (that can be transcribed from the opposite strand in the case of asRNAs or from somewhere else in the genome for the sRNAs) and hence altering their secondary structure, which might have consequences regarding their expression (ribosome accessibility) or stability (RNase accessibility). The few examples that have been analyzed in detail in *H. pylori* will be presented below.

### Antisense RNA-Mediated Regulation of Gene Expression

#### Regulation of Type I TA Systems

Toxin-antitoxin (TA) systems are genetic modules that are widespread in prokaryotes. They encode a protein toxin and a cognate antitoxin that prevents the activity or expression of the toxin. The type I TA systems, in which the antitoxin is an asRNA, are highly represented on the *H. pylori* chromosome and several are strongly expressed (Sharma et al., 2010; Masachis and Darfeuille, 2019). Among them, the type I TA *aapA1*-*isoA1* system has been extensively studied (Arnion et al., 2017; El Mortaji et al., 2020). The corresponding AapA1 toxin is a 30 aa-long hydrophobic peptide that, upon expression, targets the inner membrane and inhibits *H. pylori* growth (El Mortaji et al., 2020; Korkut et al., 2020). We have recently shown that this toxin triggers a morphological transition of *H. pylori* from its typical helical shape to round coccoid cells, that have been observed in response to stress as well as in human biopsies (El Mortaji et al., 2020). Our characterization of the toxin-induced coccoids suggests that they are viable cells that could correspond to dormant bacteria and might be responsible for *H. pylori* infections refractory to treatment.

Under normal growth conditions, different mechanisms that prevent the expression of the AapA1 toxin have been revealed (Arnion et al., 2017). Folding of the *aapA1* full length transcript prevents its translation, unless its 3′-end is processed. This active *aapA1* structure allows the formation of an extended duplex with the *isoA1* asRNA that is degraded by RNase III, thus preventing AapA1 translation (Arnion et al., 2017; Figure 2A). The expression of AapA1 occurs under conditions that reduce the activity of the *isoA1* promoter, namely oxidative stress and hence triggers *H. pylori* morphological transition (El Mortaji et al., 2020). Thus, the *isoA1* asRNA is involved in regulating the transition of *H. pylori* cells into dormant forms that likely play an important role during colonization.

In another TA system from this family, *aapA3*-*isoA3*, the expression of the toxin is regulated by the formation of metastable structures in the *aapA3* mRNA that transiently form during transcription and result in sequestration of the Shine-Dalgarno sequence, thereby also preventing unwanted toxin synthesis (Masachis et al., 2019).

#### Regulation of the Expression of the *ureAB* Operon by an asRNA

Urease is a major colonization factor for *H. pylori*. This nickel metalloenzyme catalyzes the hydrolysis of urea into ammonia and bicarbonate (de Reuse et al., 2013). Both compounds allow *H. pylori* to buffer its cytoplasm and thus resist the extremely low pH of its unique niche, the human stomach. Large amounts of urease are produced by *H. pylori*. However, its synthesis requires regulation to avoid a toxic alkalinization of the cytoplasm. Urease of *H. pylori* is composed of two structural units, UreA and UreB. These proteins are expressed from an operon that is adjacent to a second one encoding the so-called urease accessory proteins required for nickel incorporation into urease. Expression of *ureAB* is positively controlled by NikR, a nickel-responsive regulator (Muller et al., 2011; Jones et al., 2018) and by a two-component system (TCS) of the OmpR-EnvZ family (Bury-Moné et al., 2004) designated ArsRS in *H. pylori* (Pflock et al., 2006). Under low pH conditions, the ArsRS system activates the urease operons ensuring increased production of this enzyme under the condition where its activity is vital. Wen et al. (2011, 2013) have identified another level of regulation of the *ureAB* operon. At neutral pH, they demonstrated the expression of a 290 nucleotides (nt)-long asRNA to *ureB* that favors the accumulation of a truncated transcript of the *ureAB* operon lacking the *ureB* 3′-end (Figure 2B). Interestingly, the expression of this asRNA is negatively regulated by the ArsRS TCS in response to acidic pH. Electrophoretic mobility shift assays (EMSA) showed that unphosphorylated ArsR regulator protein indeed binds to the *ureB*-asRNA promoter region. They determined that the mechanism at play is base-pairing of the antisense 5′*ureB*-asRNA with the *ureAB* transcript and subsequent transcription termination of the sense *ureAB* mRNA that causes diminished urease production. These data highlight a dual control of urease production that is adjusted to the pH of the environment encountered by *H. pylori*, with activation of urease expression at acidic pH and repression at neutral pH.

#### Other Antisense RNAs

Another example concerns rRNA maturation in *H. pylori*. A strongly expressed asRNA overlapping the leader region of the 23S-5S rRNA precursor has been identified and found to be conserved (Sharma et al., 2010; Iost et al., 2019). It was demonstrated that this asRNA interacts with an rRNA precursor, forming an intermolecular complex that is cleaved by RNase III. This pairing induces further specific cleavages of the rRNA precursor and the degradation of the 23S asRNA. Whether this asRNA plays a regulatory function in rRNA maturation is still unclear as it is dispensable for *H. pylori* growth and for proper rRNA maturation under laboratory conditions. However, it might have a fine-tuning function by facilitating the degradation of processed fragments or a quality control role by favoring appropriate rRNA folding, that might become more evident under different growth conditions (Iost et al., 2019).

Other asRNAs were first identified *in silico* and then found to be expressed. They were predicted to target the flagellar motor switch gene (*fliM*) and fumarase (*fumC*), potentially regulating the expression of these genes (Xiao et al., 2009).

### *Trans-*Acting Regulatory sRNAs of *H. pylori*

#### RepG, A sRNA That Targets Simple Repeat Sequences (SRRs)

Besides transcriptional regulators, gene expression can be modulated by variations in the length of repeated nucleotide sequences, the “simple sequence repeats” (SSRs), located in their 5′-UTR that modify the stability or translation of the mRNA; or located in the promoter region, affecting the spacing of promoter elements or transcription factors binding sites. Phase variation of SSRs is due to slipped strand mispairing during replication and is used by many pathogens as an adaptive mechanism in which the most favorable expression is selected. Genes encoding bacterial surface structures or DNA Restriction-Modification enzymes are among the most frequently regulated by SSRs. The group of Sharma et al. (2010) identified and characterized the first sRNA that mediates post-transcriptional regulation by targeting a G-repeat stretch that is located in the 5′-UTR of the bicistronic *tlpB*-*hp0102* operon (Pernitzsch et al., 2014, 2021; Figure 2C). This operon encodes the TlpB chemotaxis receptor and the HP0102 protein that was shown to function as a glycosyltransferase involved in lipopolysaccharide (LPS) O-chain biosynthesis (Pernitzsch et al., 2021). The precise fucosyltransferase activity of HP0102 was shown (Li et al., 2019). The sRNA designated RepG (REgulator of Polymeric G-repeats) is highly conserved in *H. pylori*. In contrast, the length of the G-repeat stretch upstream *tlpB*-*hp0102* is highly variable among *H. pylori* strains and in sequential isolates from human patients, indicating that this region indeed undergoes phase variation during infection. Direct binding and base pairing of RepG to the 5′-UTR SSR region was demonstrated. Most interestingly, they showed that depending on the length of the G repeat, the binding of RepG can either mediate activation or repression of the *tlpB*-*hp0102* operon and that this regulation acts at the translational level (Pernitzsch et al., 2014, 2021). In a recent publication, in collaboration with our group (Pernitzsch et al., 2021), the HP0102 glycosyltransferase was shown to be essential for colonization of the mouse model by *H. pylori* and to modulate LPS O-chain synthesis. The gradual modulation of *H. pylori* LPS through RepG regulation impacts bacterial resistance to membrane-targeting antibiotics and the exposure of Lewis antigens that contribute to the host immune recognition of this pathogen. In conclusion, these studies establish an original mechanism of post-transcriptional regulation by RepG, a *trans*-acting sRNA, that mediates a gradual rather than ON/OFF switch of the expression of its targets enabling *H. pylori* to adapt to its host.

#### NikS/HPnc4160, A sRNA Regulating Major Virulence Factors in *H. pylori*

Two recent publications report the characterization of a sRNA (HPnc4160) that regulates the expression of major *H. pylori* virulence factors (Eisenbart et al., 2020; Kinoshita-Daitoku et al., 2021). HPnc4160 was previously identified as a highly transcribed sRNA (IsoB) expressed from the opposite strand of a poorly expressed small ORF (AapB) as a part of a probably “degenerated” type I TA system (Sharma et al., 2010; Vannini et al., 2017). HPnc4160 is strongly conserved in *H. pylori* and harbors a length-variable T stretch upstream its −10 promoter sequence. The expression of HPnc4160 was shown to be repressed by NikR in response to nickel (Eisenbart et al., 2020) through direct binding of this transcriptional regulator to the sRNA promoter (Vannini et al., 2017). Therefore, HPnc4160 was renamed NikS (Eisenbart et al., 2020). The T stretch preceding the HPnc4160/NikS sRNA was variable in length in strains isolated from patients or during Mongolian gerbil colonization (Eisenbart et al., 2020; Kinoshita-Daitoku et al., 2021); this length variation affects the sRNA expression. Both studies identified the targets of this sRNA by mRNA and protein expression analysis. Genes encoding major virulence and colonization factors were found to be targeted by HPnc4160/NikS, [five in Eisenbart et al. (2020) and eight in Kinoshita-Daitoku et al. (2021); see Table 1]. Both studies identified *cagA*, encoding the oncoprotein CagA, as a target and found that the expression of several outer membrane proteins, some being potential adhesins, is targeted. Eisenbart et al. identified in addition *vacA*, encoding the vacuolating cytotoxin, as being directly regulated by HPnc4160/NikS. Post-transcriptional repression by HPnc4160/NikS base pairing to five mRNA leaders was demonstrated, which results in translation inhibition (Eisenbart et al., 2020). *In vitro* EMSA and structure probing analysis validated the direct HPnc4160/NikS interaction with several of its targets (Eisenbart et al., 2020; Kinoshita-Daitoku et al., 2021). However, the two publications present divergent data on the targeting site in *cagA*, in the 5′ UTR region for Eisenbart et al. (2020) and intragenic for Kinoshita-Daitoku et al. (2021), which implies different underlying regulatory mechanisms. Both studies validate that the control of *cagA* expression by HPnc4160/NikS indeed impacts *H. pylori* pathogenicity. *In vitro* infection of different cell types by *H. pylori* revealed that HPnc4160/NikS decreases CagA-dependent bacterial internalization, reduces cell colonization and epithelial barrier disruption (Eisenbart et al., 2020) and also decreases the level of phosphorylated CagA, IL8 production and the associated CagA-induced hummingbird phenotype (Kinoshita-Daitoku et al., 2021). During mouse colonization by *H. pylori*, the number of repeats of HPnc4160/NikS varies with a trend to expansion while a deletion of this sRNA favors short term colonization. The selective pressure that drives these variations *in vivo* still needs to be identified. Finally, the T-repeats were found to be significantly longer in strains isolated from patients with gastric cancer than in “non-cancer” strains. In conclusion, HPnc4160/NikS is a sRNA acting as a master regulator of the adaptation of *H. pylori* to the colonization of its host.

#### CncR1, A sRNA Regulating Motility and Adhesion to Host Cells

The *H. pylori* cag pathogenicity island (*cag* PAI) is a 40 kb DNA element that encodes a type IV secretion system. The *cag* PAI is a major virulence determinant of *H. pylori* that allows for the delivery of bacterial effector molecules into host gastric epithelial cells, in particular the oncoprotein CagA encoded within the *cag* PAI.

Both groups of Sharma et al. (2010) and Vannini et al. (2014) have identified, within the *cag* PAI, a ncRNA expressed within the 5′-UTR of *cagP*. This 213 nt-long sRNA is abundant and conserved in *H. pylori*. It was designated HPnc2630 in strain 26695 (Sharma et al., 2010) and was renamed CncR1 for “*cag* non-coding RNA 1” in strain G27 where it was characterized (Vannini et al., 2016). Vannini et al. (2016) showed that the expression of the CncR1 sRNA is directed by the *cagP* promoter and regulated as a function of growth through direct binding of the essential orphan response regulator HsrA (HP1043). Transcriptomic analysis of a *ΔcncR1* mutant identified 71 deregulated genes. Enrichment in downregulated genes related to host-pathogen interactions was observed in addition to upregulation of genes involved in the assembly and regulation of the flagellar apparatus including FliK, a flagellar hook-length control protein. They validated that CncR1 negatively regulates *H. pylori* motility functions. Using EMSA and RNase T1 foot-printing experiments, direct targeting of the sRNA on different regions of the *fliK* mRNA was demonstrated (Figure 2C). Finally, CncR1 is required for full bacterial adhesion to host cells. In conclusion, the CncR1 sRNA modulates *H. pylori* virulence through opposite effects on motility and adhesion which might be relevant to signals related to growth phase or bacterial density where bacteria move to a new colonization site.

## Conclusion

Relatively few studies have been published on riboregulation in *H. pylori*. However, the established properties make it a fascinating microorganism to study. The huge number of genes for which a *cis*-asRNA is expressed is still intriguing, some of them certainly have regulatory roles on the expression of the complementary mRNA but many RNAs that are produced by pervasive transcription may have other functions. The post-transcriptional mode of gene regulation where a sRNA targets homopolymeric repeats within 5′-UTR is original. This mechanism and other variations linking RNA-mediated control and SSRs are most probably widespread in *H. pylori* and will certainly be found in other bacteria in the future. In *H. pylori*, RepG has other targets, several antisense TSSs were shown to overlap SSRs and some sRNA candidates have internal repeats (Sharma et al., 2010; Pernitzsch et al., 2014, 2021). These data, together with what is known about the SSR-controlled NikS/HPnc4160 sRNA, reveal that bacterial phase variation, which is associated to host adaptation, impacts gene expression at several levels. Finally, since no RNA chaperone facilitating sRNA-mRNA base-paring has been reported so far in *H. pylori*, the question of discrimination of the targets, in particular those with low complexity, such as SSRs, remains open.

## Author Contributions

Both authors wrote and edited the manuscript, contributed to the article and approved the submitted version.

## Conflict of Interest

The authors declare that the research was conducted in the absence of any commercial or financial relationships that could be construed as a potential conflict of interest.

## Footnotes

1 http://www.imib-wuerzburg.de/research/hpylori/
